# Guardian of myelin and neural Integrity: *foxo1a* through *slc7a11* mitigating oxidative damage in myelin

**DOI:** 10.1016/j.redox.2025.103763

**Published:** 2025-07-12

**Authors:** Yinjie Zhao, Zikang Li, Weiqun Lu

**Affiliations:** a National Demonstration Center for Experimental Fisheries Science Education (Shanghai Ocean University), Shanghai, 201306, China; b International Research Center for Marine Biosciences (Shanghai Ocean University), Ministry of Science and Technology, Shanghai, 201306, China; c Key Laboratory of Exploration and Utilization of Aquatic Genetic Resources (Shanghai Ocean University), Ministry of Education, Shanghai, 201306, China

**Keywords:** Myelin, *foxo1a*, Antioxidant defense, Ferroptosis, Jawed vertebrates

## Abstract

The emergence of myelin marks an evolutionary leap from jawless to jawed vertebrates. Although myelin's role in promoting rapid neural signal transmission and brain complexity is known, its neuroprotective mechanisms in complex signal transmission remain unclear. This study identifies the critical FoxO gene family member, *foxo1a*, as essential to the evolution of jawed vertebrates by comparing divergence times and gene family heterogeneity between jawless and jawed vertebrates. We found that *foxo1a* is located in zebrafish oligodendrocytes and myelin, playing a key antioxidant protective role. Specifically, we found that knocking out the *foxo1a* gene leads to abnormal myelin development in the central nervous system of zebrafish, a reduction in oligodendrocytes, astrocytes, and myelin markers, and induces freezing behavior. Further research revealed that this is related to oxidative stress responses and ferroptosis in the central nervous system of zebrafish following the deficiency of the *foxo1a* gene. Mechanistically, we discovered that *foxo1a* is involved in regulating oxidative stress responses and iron homeostasis in the central nervous system by directly regulating the promoter activity of the *slc7a11* gene. In terms of application, we found that exogenous supplementation of *foxo1a* can exert antioxidant protective effects in a copper sulfate-induced myelin damage model. More importantly, we found a parallelism of the *foxo1a-slc7a11* axis in both zebrafish and human cells, suggesting that the *foxo1a-slc7a11* axis might be an evolutionarily conserved neural defense strategy in jawed vertebrates. In conclusion, our study elucidates the critical role of *foxo1a* in maintaining antioxidant homeostasis in the central nervous system and provides new insights into the adaptive evolution of the central nervous system in jawed vertebrates.

## 1 Introduction

The formation and maintenance of species diversity constitute fundamental concerns in the fields of evolutionary biology and ecology. Within ecosystems, biodiversity encompasses not only interspecies diversity but also genetic and functional trait diversity. These diversities function at the community level through eco-evolutionary feedback mechanisms [1,2]. Genomic alterations are not merely the result of random replication errors; rather, they are likely influenced by the evolutionary needs of species [3]. The transition between the Ordovician and Silurian periods holds critical significance in the evolution of jawed vertebrates [4]. The characteristics of this period include severe climate changes and environmental degradation, such as global warming and ocean deoxygenation, leading to the mass extinction of many species. After this, the complexity of Paleozoic faunal assemblages increased, marking the turnover of marine biota during the Ordovician and Silurian periods [[5], [6], [7]]. The emergence of jawed vertebrates represents a pivotal event [4]. Compared to jawless vertebrates, the evolution of jawed vertebrates not only facilitated active feeding and movement but also significantly enhanced environmental adaptability, thereby greatly augmenting the overall evolutionary potential of vertebrates [8,9]. This evolutionary advancement is largely attributed to the presence of myelin in jawed vertebrates, a feature that propelled their advanced adaptive evolution [10].

Myelin, a densely layered membranous structure encasing neuronal axons, constitutes an evolutionary advancement that has allowed vertebrates to develop more complex behavioral patterns and physiological functions, such as rapid responses and enhanced sensory abilities. This is accomplished through the facilitation of action potential conduction, the acceleration of neural network signal integration, and the enhancement of the efficiency and precision of neural signal transmission [11,12]. Importantly, myelin enhances signal transmission speed without necessitating an increase in axon diameter, thus accommodating the multitude of axons required for the evolution of intricate nervous systems [[13], [14], [15]]. From the perspective of energy metabolism, jawed vertebrates generally exhibit higher metabolic rates compared to their jawless counterparts and possess more complex nervous systems [16,17]. Under standard physiological conditions, neurons primarily generate adenosine triphosphate (ATP) through mitochondrial oxidative phosphorylation [18]. However, this process is associated with the production of reactive oxygen species (ROS) due to electron leakage within the mitochondrial respiratory chain [19], leading to a greater accumulation of ROS in jawed vertebrates than in jawless vertebrates. Notably, jawed vertebrates appear to possess effective mechanisms for mitigating the risk of excessive ROS accumulation and the resultant oxidative damage [20,21]. Nonetheless, the specific mechanisms by which myelin and neurons are safeguarded against oxidative damage during efficient signal transmission remain a significant enigma in the evolutionary biology of jawed vertebrates.

The Forkhead box O (FoxO) transcription factor family, a gene family highly conserved throughout evolution, is ubiquitously expressed in nearly all vertebrates. This family regulates essential biological processes, including cell cycle control, metabolism, immune regulation, proliferation, differentiation, and apoptosis [[22], [23], [24]]. Additionally, numerous studies have found that members of the FoxO family play an important role in antioxidant protection. For instance, research involving A14, C2C12, and 293T cell lines has shown that FoxO4 modulates redox reactions. In these studies, oxidative stress induced by hydrogen peroxide activates the small GTPase Ral, which in turn activates c-Jun N-terminal kinase (JNK)-dependent FoxO4. This activation enhances cellular protection against oxidative stress by upregulating manganese superoxide dismutase (MnSOD) and catalase [25]. In chondrocytes, the siRNA-mediated downregulation of FoxO1 and FoxO3 significantly increased cell death susceptibility to *tert*-butyl hydroperoxide, which was associated with decreased levels of antioxidant and autophagy-related proteins [26]. Furthermore, FoxO6 has been implicated in the regulation of oxidative stress within skin cells. Specifically, adenovirus-mediated knockdown or activation of FoxO6 in B16F10 cells resulted in the upregulation or downregulation of antioxidant gene expression, respectively [27]. This suggests that the FoxO family plays a crucial role in maintaining cellular redox homeostasis. Notably, a recent study has demonstrated that FoxO1 immunohistochemical signals are specifically localized to Schwann cells in the peripheral nervous system [28]. Given that Schwann cells are responsible for myelin formation in this system, it is hypothesized that FoxO1 may play a role in the antioxidant protection of myelin within the nervous system. However, up until now, there has been no relevant research supporting the molecular evidence that FoxO1 plays a neuroprotective role in myelin and neurons. This is largely due to the lethal phenotype resulting from the endogenous deletion of the FoxO1 gene in mammals, which leads to a lack of genetic evidence demonstrating the functional role of FoxO1 in antioxidant protection in myelin and neurons [29]. Moreover, it is unknown whether the antioxidant protective function of FoxO1 is an evolutionarily conserved mechanism in jawed vertebrates, including lower animals.

The development of myelin during vertebrate evolution marks a critical transition from jawless to jawed vertebrates. Although the functional benefits of myelin in promoting rapid neural signal transmission and enhancing brain complexity are well-established [30,31], the neuroprotective mechanisms of myelin during complex signal transmission are not yet fully understood. Here, we identify the FoxOs gene family member *foxo1a* as a critical factor in the adaptive evolution of jawed vertebrates by comparing evolutionary divergence times and gene family heterogeneity between jawless and jawed vertebrates. Using a zebrafish model, we found that *foxo1a* is localized in oligodendrocytes and myelin, where it plays a vital role in antioxidant protection. Deletion of *foxo1a* leads to loss of a compact myelin sheath of axons, reduced oligodendrocyte and myelin markers, aberrant astrocyte gene transcription, and neurological behavioral defects, accompanied by heightened oxidative stress and ferroptosis in the brain. Conversely, *foxo1a* overexpression rescues these damages and alleviates oxidative injury in a CuSO_4_-induced myelin damage model. Mechanistically, *foxo1a* interacts with FoxOs binding sites in the promoter regions of *slc7a11*, regulating oxidative stress and iron signaling. It is significant to note that the amino acid sequence and protein domains of *foxo1a* are highly conserved among jawed vertebrates, with its evolutionary origin aligning with the emergence of this group. This conservation suggests that *foxo1a* may possess neuroprotective functions across different species. Our findings reveal that *foxo1a* is essential for maintaining antioxidant homeostasis in the nervous system and highlight its pivotal role in the adaptive evolution of jawed vertebrates.

## 2 Results

1. Divergence time phylogenetic tree and gene family expansion

**Fig. 1:**
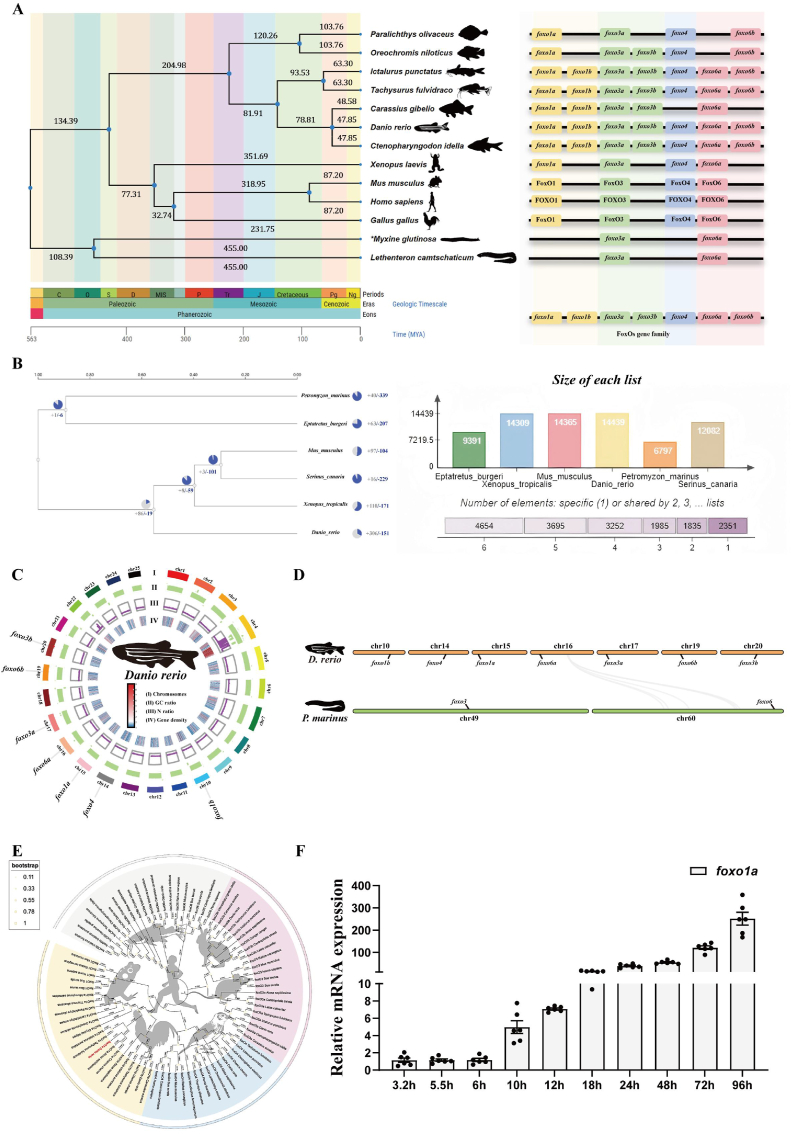
Phylogenetic tree of divergence times and expansion of gene families. A: Evolutionary analysis of FoxO divergence times, showing the distribution of FoxO gene families in different species. *foxo1a* is highly conserved among jawed vertebrates, and its first appearance in the vertebrate genome coincides with the emergence of jawed vertebrates. B: Analysis of gene family expansion, with a significant expansion of the FoxO gene family. C: Distribution of FoxO gene family members on zebrafish chromosomes. D: Synteny analysis between zebrafish and lamprey. E: Phylogenetic tree of the FoxO family proteins. F: Early expression of the zebrafish *foxo1a* gene.

To investigate the origin of the FoxOs gene family, a phylogenetic tree was constructed using single-copy genes shared among species. Divergence times were estimated, and the sequences of the FoxOs gene family from vertebrates were collected from the NCBI database. Results showed that FoxO3a and FoxO6a existed in jawless vertebrates. Other members of the FoxOs family were identified in jawed vertebrates, among which *foxo1a* was highly conserved in jawed vertebrates. The time of its initial incorporation into the vertebrate genome coincided with the emergence of jawed vertebrates (Fig. 1A). Changes in gene family copy numbers play a critical role in facilitating phenotypic diversification and evolutionary adaptation to the environment [32]. We further compared gene families across 14 vertebrates and identified 100 expanded gene families in the genomes of jawed vertebrates. Among them, we found that the Forkhead Box transcription factor (FoxOs) gene family in jawed vertebrates was significantly expanded (Fig. 1B). In zebrafish, the FoxO family was found to consist of seven subtypes (Fig. 1C). This result was validated through synteny analysis of representative species. The genomes of jawed vertebrates were annotated with the FoxO1, FoxO3, FoxO4, and FoxO6 subfamilies, while only FoxO3 and FoxO6 were annotated in the genomes of invertebrates (Fig. 1D). The phylogenetic tree revealed that all Forkhead transcription factor genes across species could be grouped into four major clades. FoxO1a and FoxO1b clustered into one clade, FoxO3a and FoxO3b clustered into another clade, FoxO4 formed a separate clade, and FoxO6a and FoxO6b clustered into a fourth clade (Fig. 1E). This indicates that FoxOs family members played an essential role in the evolution of jawed vertebrates, among which *foxo1a* may be particularly significant.

2. Spatiotemporal expression of the foxo1a gene in zebrafish

**Fig. 2:**
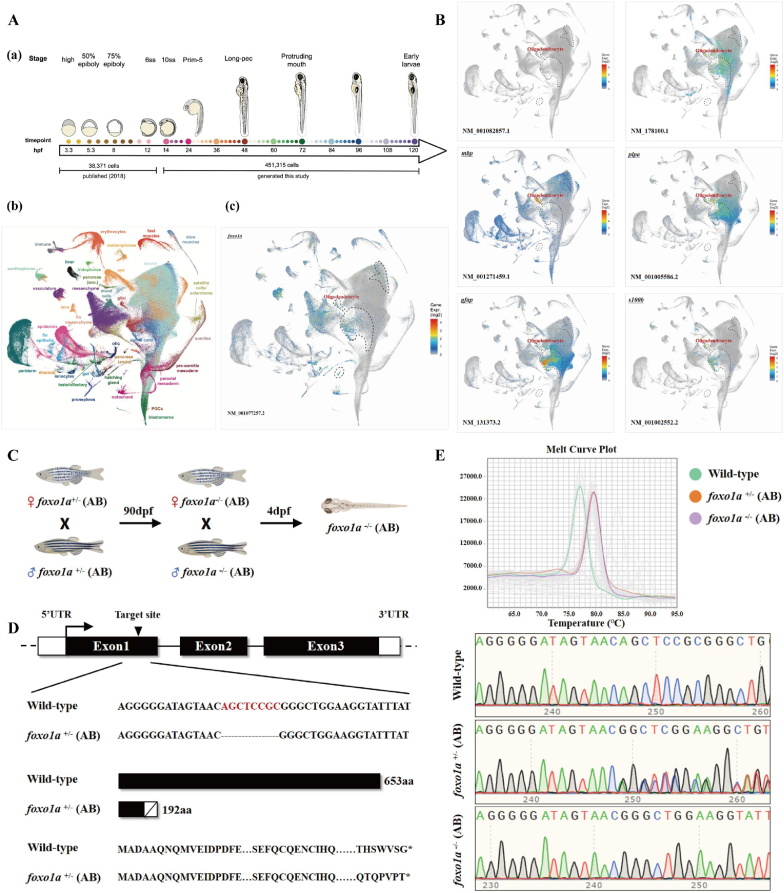
Spatiotemporal Expression and mutant Establishment of Zebrafish *foxo1a* A: Single-cell RNA sequencing data of early zebrafish development, (a) high temporal resolution single-cell RNAseq time course, covering embryogenesis and early larval development. (b) UMAP projection of single-cell transcriptomes colored by selected major tissues. (c) *foxo1a* single-cell RNA data during early development. B: Early developmental single-cell data of *foxo1b*, *olig2*, *mbp*, *plpa*, *gfap* and *s1*00b. C: Schematic diagram of *foxo1a* mutant establishment. D: Schematic diagram of the *foxo1a* mutant target. The deletion of 8bp (AGCTCCGC) in the first exon results in a frameshift mutation. E: HRMA of the *foxo1a* gene, sequencing chromatograms for genotyping wild-type, *foxo1a* heterozygous, and homozygous mutants.

To investigate the physiological function of *foxo1a* in jawed vertebrates, we selected the model organism zebrafish as the subject of study. First, we observed the expression pattern of the *foxo1a* gene during the early developmental stages of zebrafish. In this study, The RT-qPCR results showed that the expression level of the *foxo1a* gene was highest at 96 hpf, relatively low during the stages prior to 10 hpf, and then gradually increased (Fig. 1F). These results suggest that *foxo1a* is a zygotic expression gene. Furthermore, to better understand the spatial expression information of *foxo1a* during early development, we reanalyzed public single-cell RNA sequencing datasets of zebrafish early development. The analysis results revealed that *foxo1a* is highly expressed in the vasculature, muscles, and nervous system, with oligodendrocytes being the primary cell type in the nervous system where *foxo1a* is strongly expressed (Fig. 2A). In addition, *foxo1b* was found to have insignificant expression in oligodendrocytes. The expression of the *foxo1a* gene in oligodendrocyte clusters significantly overlapped with markers of oligodendrocyte precursor cells (), oligodendrocyte markers *(mbp, plpa*), and astrocyte markers (*gfap, s100b*) (Fig. 2B). This indicates that *foxo1a* may play an important role in the development and differentiation of oligodendrocytes.

3. Construction and Validation of foxo1a Gene Knockout Mutant Zebrafish Line

The *foxo1a*^+/−^ genotype zebrafish (CZRC catalog number: CZ1342) is a heterozygous mutant line carrying the ihb390 allele, purchased from the China Zebrafish Resource Center. This mutation was induced using CRISPR-Cas9 on an AB genetic background. Based on this, we performed inbreeding and obtained a homozygous mutant zebrafish line (Fig. 2C). Additionally, the *foxo1a* gene target site is located in exon 1 (Fig. 2D), and there is a deletion of 8 base pairs (i.e., AGCTCCGC) in exon 1 of the homozygous mutant zebrafish, causing a frameshift mutation (amino acid deletion schematic) (Fig. 2D). Finally, we used HRMA and DNA sequencing technology to display the genotyping results of wild-type, *foxo1a* heterozygous, and homozygous mutant zebrafish (Fig. 2E).

4. Deletion of foxo1a gene leads to abnormal development of telencephalic oligodendrocytes and myelination in zebrafish.

**Fig. 3:**
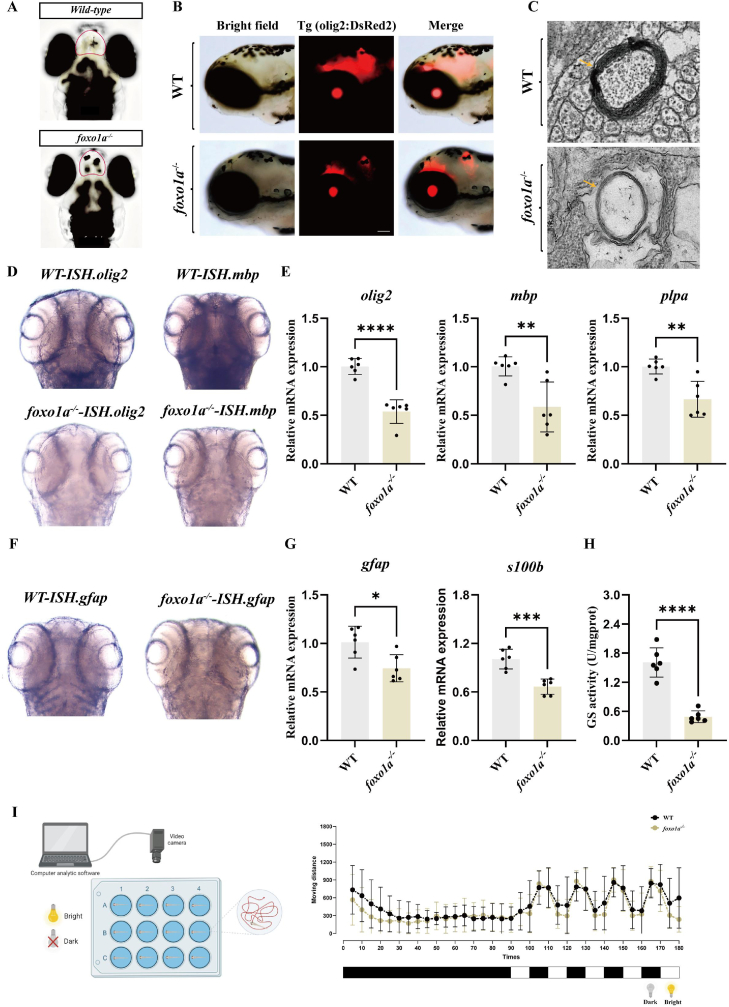
Mutant of the *foxo1a* gene leads to abnormal development of the telencephalon, oligodendrocytes, and myelin in zebrafish, as well as abnormal development of astrocytes and behavioral abnormalities. A: Dorsal view of the brains of control and *foxo1a*^*−/−*^ larvae, the red coil indicates the position of the telencephalon. B: Expression of *olig2* fluorescent protein in the brains of control and *foxo1a*^*−/−*^ groups, with reduced fluorescence in the *foxo1a*^*−/−*^ group, scale bar = 100 μm C: Representative transmission electron micrographs of myelin in control and *foxo1a*^*−/−*^ larvae, the yellow arrow indicates the location of the myelin, scale bar = 100 nm D: In situ hybridization of *olig2* and *mbp* in control and *foxo1a*^*−/−*^ groups. E: qRT-PCR analysis of *olig2, mbp*, and *plpa* gene expression in oligodendrocytes of control and *foxo1a*^*−/−*^ zebrafish larvae, N = 6, *t*-test, ∗p < 0.05, ∗∗p < 0.01, ∗∗∗p < 0.001, ∗∗∗∗p < 0.0001 F: In situ hybridization of *gfap* in control and *foxo1a*^*−/−*^ groups. G: Expression of *gfap* and *s100b* genes in astrocytes of control and *foxo1a*^*−/−*^ zebrafish larvae.,N = 6, *t*-test, ∗p < 0.05, ∗∗p < 0.01, ∗∗∗p < 0.001, ∗∗∗∗p < 0.0001 H: Comparison of glutamine synthetase activity in control and *foxo1a*^*−/−*^ zebrafish larvae, N = 6, *t*-test, ∗p < 0.05, ∗∗p < 0.01, ∗∗∗p < 0.001, ∗∗∗∗p < 0.0001 I: Schematic of the light-dark alternation behavior experiment, where the Viewpoint tool observes zebrafish for 90 min under normal daylight, followed by minute-by-minute light-dark alternations, and records the distance moved by control and *foxo1a*^*−/−*^ zebrafish larvae every 10 min.

After obtaining *foxo1a* deletion mutants, this study first conducted a preliminary observation of the phenotype of mutant zebrafish to explore the impact of the *foxo1a* gene on the physiological functions of zebrafish. It was found that the telencephalic size of *foxo1a* gene-deficient zebrafish larvae was significantly reduced compared to wild-type zebrafish larvae (Fig. 3A). The telencephalic is the highest-level region of the brain and includes functional areas responsible for auditory, olfactory, and motor functions. Interestingly, the oligodendrocyte biomarker is specifically expressed in the telencephalic, which is also the main cell type with high *foxo1a* expression in our previously analyzed single-cell dataset. Therefore, we crossed *foxo1a* gene knockout zebrafish with transgenic zebrafish Tg (olig2:DsRed2) to visualize the effect of *foxo1a* gene deficiency on zebrafish oligodendrocytes. The results showed that the loss of the *foxo1a* gene led to a significant reduction in the fluorescence intensity of the oligodendrocyte marker olig2 in the telencephalic of zebrafish (Fig. 3B). This indicates that the *foxo1a* gene is crucial for the normal development of oligodendrocytes in the zebrafish telencephalic.

Oligodendrocytes are the primary cells responsible for forming myelin sheaths [33]. To explore whether the abnormalities in oligodendrocytes in mutants further resulted in changes to the myelin sheath, transmission electron microscopy was performed on 4 dpf zebrafish larvae. The results indicate that the myelin structure in the brains of wild-type zebrafish is intact and dense, while in zebrafish without *foxo1a*, the myelin boundaries in the brain are unclear, the myelin structure is loose and thinner, the layered structure is severely degenerated, thickness is reduced, and a compact myelin sheath cannot be formed around the axons. (Fig. 3C). Additionally, this study conducted ISH experiments targeting oligodendrocyte transcription factor and myelin basic protein gene *mbp* in 4 dpf larvae (Fig. 3D) and RT-qPCR experiments on oligodendrocyte marker genes (Fig. 3E). The experimental results showed that in 4 dpf *foxo1a* gene-deficient larvae, the expression levels of *olig2* and *mbp* in the brain were lower than those in wild-type zebrafish larvae. Moreover, the expression of oligodendrocyte marker genes*, mbp*, and *plpa* was significantly downregulated, with statistical differences observed. Based on these comprehensive results, it can be inferred that there may be damage to oligodendrocytes and myelin sheaths in the brains of *foxo1a* gene-deficient zebrafish larvae. However, the underlying causes of these abnormalities still require further experimental investigation.

5. The deletion of foxo1a leads to impaired astrocytes in the zebrafish telencephalic and induces a freezing behavior in zebrafish.

During neural development, oligodendrocytes not only form myelin sheaths but can also differentiate into astrocytes [34]. Therefore, we also investigated whether astrocytes in the zebrafish brain exhibit abnormalities following the deletion of the *foxo1a* gene. In this study, we performed ISH experiments for the astrocyte marker gene *gfap* (Fig. 3F) and RT-qPCR for astrocyte marker genes (Fig. 3G) on 4 dpf larvae. The experimental results showed that in 4 dpf *foxo1a* gene-deficient zebrafish larvae, the expression level of *gfap* in the brain was lower than that in wild-type larvae, and the expression of astrocyte marker genes *gfap* and *s100b* was significantly reduced, consistent with the ISH results. Furthermore, this study measured glutamine synthetase activity (Fig. 3H) and conducted light-dark transition behavioral experiments on 4 dpf larvae. The results showed that *foxo1a* gene-deficient zebrafish exhibited reduced glutamine synthetase activity in the brain and displayed pronounced freezing behavior (Fig. 3I). Therefore, the deletion of the *foxo1a* gene in zebrafish larvae leads to a reduced number of oligodendrocyte precursor cells in the brain, which in turn results in a decrease in differentiated astrocytes. This condition may cause a decrease in glutamine synthetase activity, leading to an imbalance of excitatory and inhibitory neurotransmitters in the brain and subsequently triggering abnormal mental behaviors. These findings further support the reliability of the data on the abnormal telencephalic phenotype.

6. The deletion of foxo1a leads to impaired antioxidant system and increased ROS accumulation in the zebrafish brain.

**Fig. 4:**
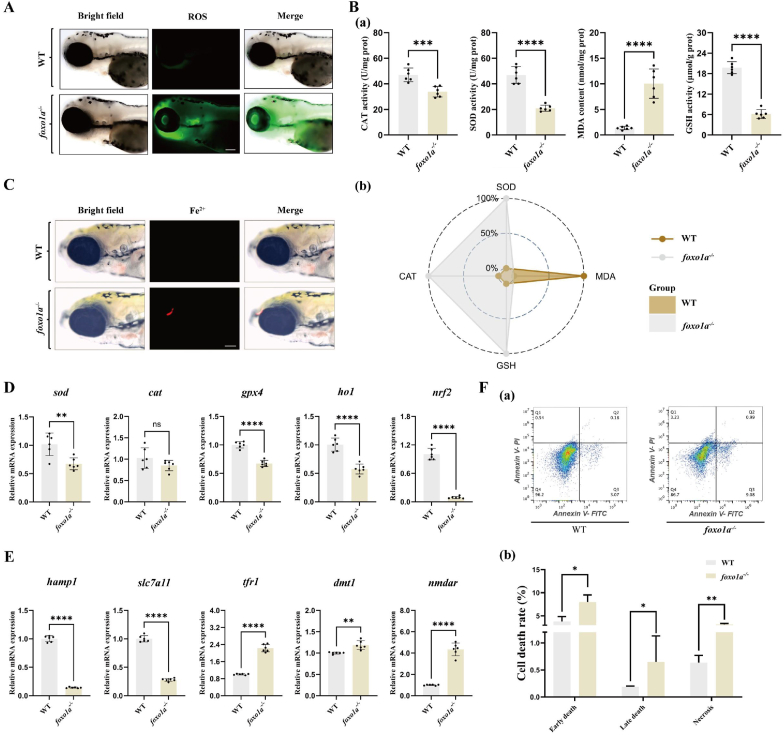
Deletion of the *foxo1a* gene leads to abnormal antioxidant systems in the zebrafish brain and ferroptosis occurs in the zebrafish brain. A: ROS fluorescence images of the brains of control group and *foxo1a*^*−/−*^ group zebrafish larvae, scale bar = 100 μm B: (a) The effect of antioxidant enzyme activity in the brain tissue of control group and *foxo1a*^*−/−*^ group zebrafish larvae, including SOD activity, CAT activity, GSH activity and MDA content. N = 6, *t*-test, ∗p < 0.05, ∗∗p < 0.01, ∗∗∗p < 0.001, ∗∗∗∗p < 0.0001. (b) IBR calculation radar chart of antioxidant enzyme activity in the brain tissue of control group and *foxo1a*^*−/−*^ group zebrafish larvae. N = 6, data are expressed as mean ± SD. C: Iron ion fluorescence images of the brains of control group and *foxo1a*^*−/−*^ group zebrafish larvae, scale bar = 100 μm. D: qRT-PCR detection of changes in oxidative stress-related mRNA expression (*sod, cat, ho1, gpx4* and *nrf2*) in the brain tissue of control group and *foxo1a*^*−/−*^ group zebrafish larvae. N = 6, *t*-test, ∗p < 0.05, ∗∗p < 0.01, ∗∗∗p < 0.001, ∗∗∗∗p < 0.0001. E: qRT-PCR detection of changes in ferroptosis-related gene mRNA expression of *hamp1, tfr1, dmt1, slc7a11* and *nmdar* in the brain tissue of control group and *foxo1a*^*−/−*^ group zebrafish larvae. N = 6, *t*-test, ∗p < 0.05, ∗∗p < 0.01, ∗∗∗p < 0.001, ∗∗∗∗p < 0.0001. F: (a) Flow cytometry detection of apoptosis in the brains of control group and *foxo1a*^*−/−*^ group zebrafish larvae. (b) The number and proportion of early apoptotic, late apoptotic, and necrotic cells in the brains of control group and *foxo1a*^*−/−*^ group zebrafish larvae, *t*-test, ∗p < 0.05, ∗∗p < 0.01.

The level of oxidative stress in the nervous system is one of the key factors affecting cell differentiation and development [35]. To investigate whether the deletion of the *foxo1a* gene, which leads to oligodendrocyte and myelin damage, is related to oxidative stress, we detected an increase in ROS levels using dihydroethidium (DHE) fluorescence (Fig. 4A). Furthermore, we examined several key antioxidant enzyme activity indicators commonly used to reflect the intensity of oxidative stress. The results showed a significant decrease in the activities of superoxide dismutase (SOD), catalase (CAT), and Glutathione (GSH), along with an increase in lipid peroxidation (MDA) levels measured by thiobarbituric acid reactive substances (Fig. 4B).

Next, to further investigate the molecular mechanisms by which the *foxo1a* gene participates in the oxidative stress response, we examined the transcription levels of genes encoding antioxidant enzymes and key transcription factors in the brains of zebrafish larvae following the deletion of the *foxo1a* gene. the mRNA expression of *sod, cat*, and *gpx4* corresponded to the changes in enzyme activities, all showing significant reductions, with SOD being the most pronounced. Additionally, the mRNA expression of *nrf2* and *ho1* was also significantly downregulated (Fig. 4D). Overall, these findings suggest that the deletion of the *foxo1a* gene leads to impaired antioxidant systems in the zebrafish brain, resulting in increased levels of oxidative stress.

7. The deletion of the foxo1a gene leads to iron ion deposition in the zebrafish brain, which in turn causes ferroptosis in the brain

Oxidative stress-induced damage is often accompanied by the occurrence of cell death [36]. We attempted to investigate whether cell death occurs in the brains of *foxo1a* gene-deficient zebrafish larvae and the mode of this cell death. Flow cytometry analysis first revealed that the number of cell deaths in the brains of *foxo1a* gene-deficient zebrafish larvae was significantly increased compared to wild-type zebrafish larvae (Fig. 4F). An increase in lipid peroxides (such as MDA) is one of the key markers of ferroptosis. Based on previous experimental results, we hypothesized that the cell death induced by *foxo1a* gene deficiency involves ferroptosis. We further used a Fe^2+^ fluorescent probe (FerroOrange) to measure the ferrous ion levels in the brains of zebrafish with different genotypes. The experimental results showed that the fluorescence signal of Fe^2+^ in the telencephalon of *foxola* gene-deficient zebrafish larvae was significantly enhanced (Fig. 4C). Subsequently, we conducted a fluorescence grayscale conversion to assess the relative Fe^2+^ content (Supplementary Fig. 1A). The results showed that the Fe^2+^ content in the telencephalon of *foxola* gene-deficient zebrafish larvae was significantly increased, indicating an imbalance in iron ion metabolism in the telencephalon of these zebrafish larvae with the *foxola* gene deficiency.

To further determine the molecular mechanisms underlying ferroptosis triggered by *foxo1a* gene deficiency, we examined the transcription levels of several ferroptosis-related genes. the deficient of the *foxo1a* gene resulted in a significant downregulation in the mRNA expression levels of ferroptosis-related genes such as *hamp1, slc7a11,* and *nmdar* in the brains of zebrafish larvae, while the mRNA expression levels of *tfr1* and *dmt1* were significantly upregulated (Fig. 4E). This indicates that the occurrence of ferroptosis due to *foxo1a* gene deficiency is associated with abnormalities in the hepcidin pathway and the glutathione synthesis pathway.

8. Exogenous supplementation of foxo1a can rescue oxidative damage in the nervous system of foxo1a gene-deficient zebrafish.

**Fig. 5:**
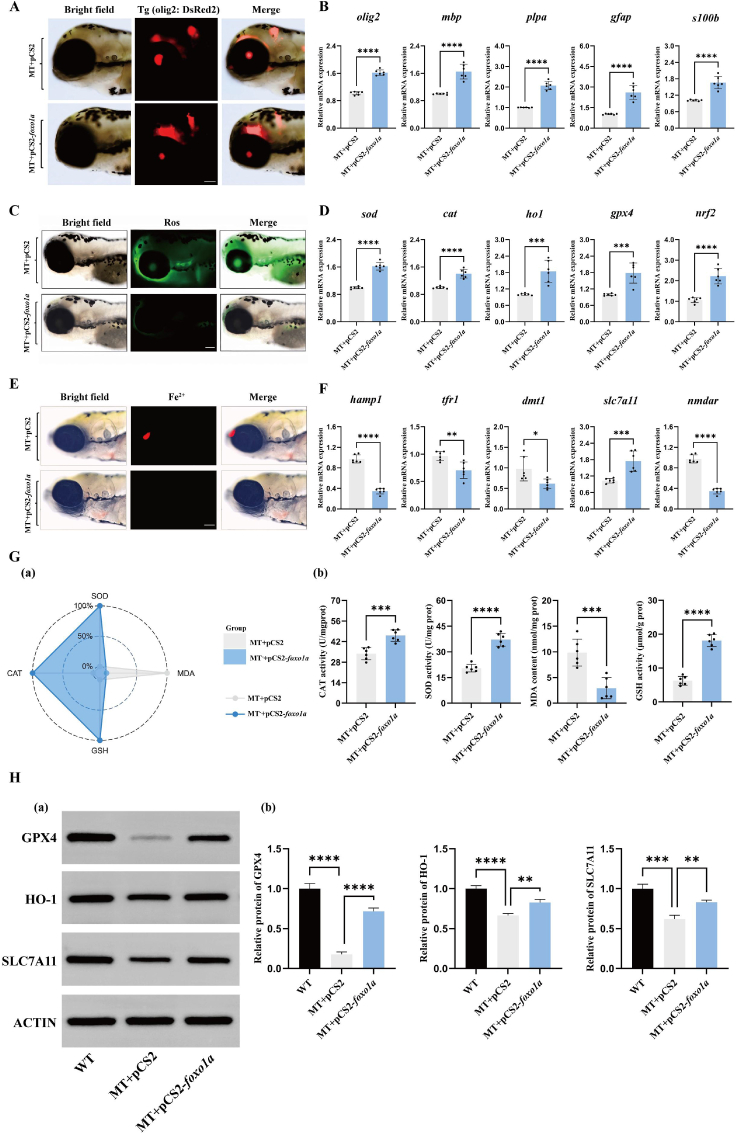
Overexpression of *foxo1a* rescues oligodendrocytes, reduces ROS levels, and alleviates ferroptosis. A: Fluorescent expression of *olig2* in the brains of zebrafish larvae receiving empty vector and overexpressing *foxo1a*, scale bar = 100 μm B: Expression of *olig2, mbp, plpa, gfap*, and *s100b* oligodendrocyte-related genes in mutants receiving empty vector and overexpressing *foxo1a,* N = 6, *t*-test, ∗p < 0.05, ∗∗p < 0.01, ∗∗∗p < 0.001, ∗∗∗∗p < 0.0001. C: ROS fluorescence in the brains of zebrafish larvae receiving empty vector and overexpressing *foxo1a*, scale bar = 100 μm. D: qRT-PCR detection of antioxidant-related gene expression (*sod, cat, ho1, gpx4* and *nrf2*) in the brain tissues of zebrafish larvae receiving empty vector and overexpressing *foxo1a*, N = 6, *t*-test, ∗p < 0.05, ∗∗p < 0.01, ∗∗∗p < 0.001, ∗∗∗∗p < 0.0001. E: Fluorescence images of iron ions in the brains of zebrafish larvae receiving empty vector and overexpressing *foxo1a*, scale bar = 100 μm F: qRT-PCR detection of changes in mRNA expression of ferroptosis-related genes (*hamp1, tfr1, dmt1, slc7a11* and *nmdar*) in the brain tissues of zebrafish larvae receiving empty vector and overexpressing *foxo1a*, N = 6, *t*-test, ∗p < 0.05, ∗∗p < 0.01, ∗∗∗p < 0.001, ∗∗∗∗p < 0.0001. G: Effects of antioxidant enzyme activity in zebrafish larvae brain tissues, (a) IBR calculated star map of antioxidant enzyme activity in the brain tissues of control group and *foxo1a*^*−/−*^ group zebrafish larvae. (b) SOD activity, CAT activity, GSH activity and MDA content. N = 6, *t*-test, ∗p < 0.05, ∗∗p < 0.01, ∗∗∗p < 0.001, ∗∗∗∗p < 0.0001. H: (a) Western blotting was used to detect changes in GPX4, HO-1, and SLC7A11 proteins in the brain tissue of the injection of the empty carrier wild-type zebrafish, the injection of the empty carrier mutant zebrafish, and the overexpression of *foxo1a* mutant zebrafish. MT represents the *foxo1a* mutant, (b) followed by normalization for relative protein quantification, oneway ANOVA with Tukey's post-hoc test, ∗p < 0.05, ∗∗p < 0.01, ∗∗∗p < 0.001, ∗∗∗∗p < 0.0001.

To eliminate false positives caused by off-target effects of gene editing, we supplemented exogenous *foxo1a* overexpressing plasmids in *foxo1a* gene-deficient zebrafish to rescue and verify abnormal phenotypes such as iron overload, oxidative stress, and nervous system damage. First, we constructed a pCS2-*foxo1a* overexpression plasmid, which was transfected into *foxo1a* gene-deficient zebrafish embryos via microinjection. On the fourth day post-fertilization, experimental observations were conducted. Compared to foxo1a gene-deficient zebrafish injected with an empty vector, those injected with the exogenous *foxo1a* overexpression plasmid showed significantly reduced damage to oligodendrocytes (Fig. 5A). Additionally, the expression of oligodendrocyte development-related marker genes *mbp, plpa, gfap,* and *s100b* was significantly upregulated (Fig. 5B). Moreover, the ROS content in the brain tissues of *foxo1a* gene-deficient zebrafish injected with the exogenous *foxo1a* overexpression plasmid was significantly reduced (Fig. 5C). Simultaneously, the expression of antioxidant-related genes *sod, cat, ho1, gpx4,* and *nrf2* was significantly upregulated (Fig. 5D). The activities of superoxide dismutase, catalase, and glutathione peroxidase 4 were also significantly increased, while lipid peroxide levels decreased (Fig. 5G). Accordingly, we also found that the Fe^2+^ fluorescence signal and relative content in the telencephalon of *foxola* gene-deficient zebrafish juveniles injected with exogenous *foxola* overexpression plasmids were significantly reduced (Fig. 5E, Supplementary Fig. S1B), and the mRNA expression of ferroptosis-related genes *hamp1, slc7a11, tfr1, dmt1,* and *nmdar* returned to normal levels (Fig. 5F). Additionally, we conducted further protein level assessments of several key genes. Immunoblotting results showed that compared to wild-type zebrafish, the protein levels of *slc7a11, gpx4,* and *ho1* were downregulated in the brain tissues of *foxo1a* gene-deficient zebrafish. Correspondingly, exogenous supplementation of *foxo1a* effectively restored the protein levels of *slc7a11, gpx4,* and *ho1* in the brain tissues of *foxo1a* gene-deficient zebrafish (Fig. 5H). This indicates that the *foxo1a* gene indeed plays an antioxidant protective role in the brain tissues of zebrafish.

9. foxo1a mediates oxidative defense in the zebrafish nervous system and prevents oligodendrocyte oxidative damage by regulating the expression of slc7a11.

**Fig. 6:**
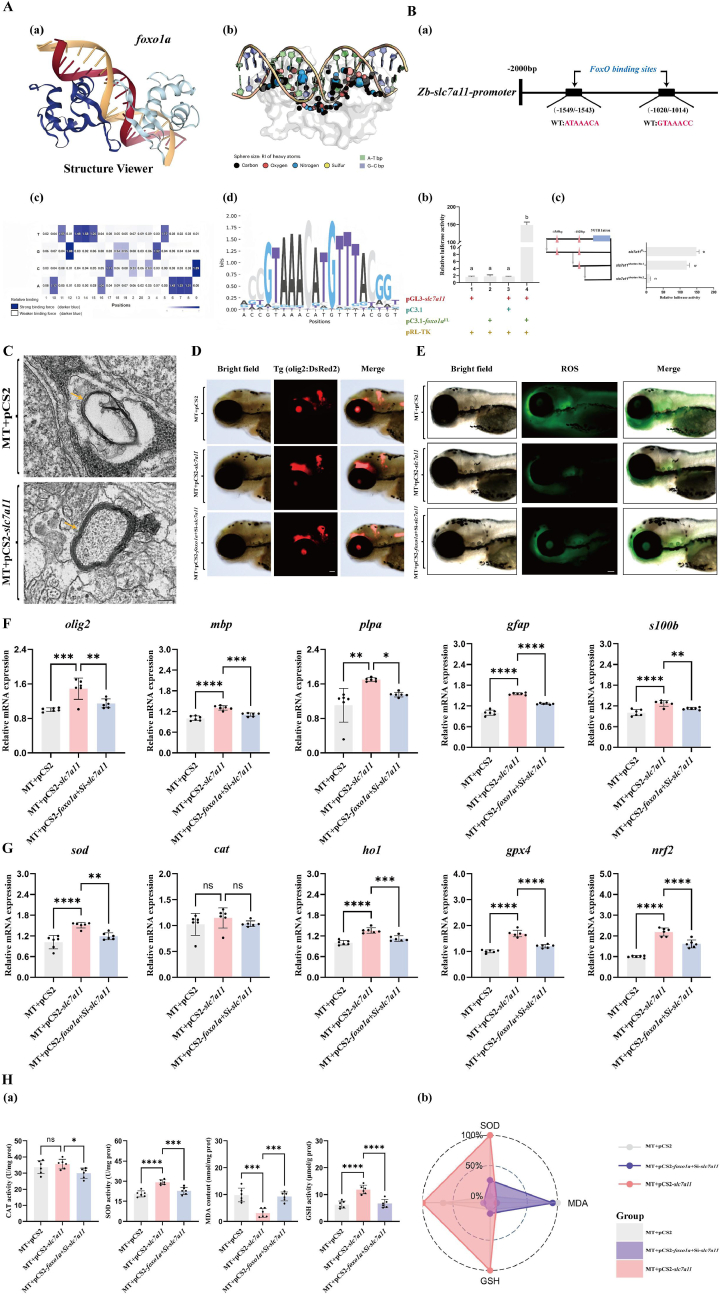
Transcription factor *foxo1a* regulates the transcription of the *slc7a11* gene, protects oligodendrocytes, enhances antioxidant capacity, and reduces ferroptosis. A: Prediction of transcription factor binding sites. (a) Schematic diagram of the binding of the *foxo1a* transcription factor to DNA structure. (b) Calculation of the relative importance (RI) score of heavy atoms within a symmetrical helix range of 5A (represented by sphere sizes: maximum 1, minimum 0) (normalized to the maximum value between atoms). (c) DeepPBS output binding specificity score. (d) Base selection intensity at the promoter sites of target genes bound by the *foxo1a* gene. Purple indicates competitive mutations where the competition is stronger than the WT competitor, while white indicates weaker competitor mutations. B: (a) Analysis diagram of the specific binding sites of *foxo1a* on the *slc7a11* gene promoter. (b) pGL3 and pC3.1 represent the circular pGL3-basic plasmid and the circular pcDNA3.1 (+) plasmid, respectively. The *slc7a11* gene promoter sequence was homologously recombined with the linearized pGL plasmid to construct the reporter plasmid pGL-*slc7a11*. The full-length CDS sequence of *foxo1a* was homologously recombined with the linearized pcDNA3.1 (+) plasmid to construct the expression plasmid pC3.1-*foxo1a*. The Rluc-expressing pRL-TK plasmid was co-transfected in each group to characterize transfection efficiency. The Arabic numerals near the x-axis indicate different treatment groups. Different letters indicate significant differences (P < 0.05). (c) Fragmentation deletion experiment on the transcriptional regulation of the *slc7a11* gene by the transcription factor *foxo1a.* Pink triangles (△) indicate the predicted binding sites of the *foxo1a* transcription factor by DeepPBS software. Three black horizontal lines represent the length of the promoter sequences in the reporter plasmid. They were obtained by homologous recombination of the fragmented pGL3-basic plasmid with different lengths of promoter sequences from the *slc7a11* gene. Different letters indicate significant differences (P < 0.05). C: Representative transmission electron microscopy images of myelin in mutant zebrafish larvae injected with empty plasmid and in mutants overexpressing slc7a11. MT represents foxo1a mutant zebrafish.Yellow arrows indicate the location of the myelin, scale bar = 100 nm. D: Injecting null mutant, overexpressing *slc7a11* mutant, overexpressing *foxo1a* mutant zebrafish then knocking down *slc7a11* again, Olig2 fluorescent expression, MT represents *foxo1a* mutant zebrafish, scale bar = 100 μm E: Injecting empty vector mutants, overexpressing *slc7a11* mutants, overexpressing *foxo1a* mutants in zebrafish followed by knockdown of *slc7a11*, ROS fluorescence expression, MT represents *foxo1a* mutant zebrafish, scale bar = 100 μm F: Injection of empty vector mutant, overexpression of *slc7a11* mutant, and overexpression of *foxo1a* mutant zebrafish followed by knockdown of *slc7a11* in larvae brain *olig2, mbp, plpa, gfap* and *s100b* oligodendrocyte-related gene expression. MT represents *foxo1a* mutant zebrafish, N = 6, oneway ANOVA with Tukey's post-hoc test, ∗p < 0.05, ∗∗p < 0.01, ∗∗∗p < 0.001, ∗∗∗∗p < 0.0001 G: Injecting empty vector mutants, overexpressing *slc7a11* mutants, overexpressing *foxo1a* mutants and then knocking down *slc7a11* in zebrafish larvae brain tissue, examining the expression of antioxidant-related genes *sod, cat, ho1, gpx4* and *nrf2.* MT represents *foxo1a* mutant zebrafish, N = 6, oneway ANOVA with Tukey's post-hoc test, ∗p < 0.05, ∗∗p < 0.01, ∗∗∗p < 0.001, ∗∗∗∗p < 0.0001. H: (a) Injection of empty vector *foxo1a* mutant, overexpression of *slc7a11* mutant, overexpression of *foxo1a* mutant followed by knockdown of *slc7a11* on antioxidant enzyme activity in zebrafish larvae brain tissue: SOD activity, CAT activity, GSH activity and MDA content. MT represents *foxo1a* mutant zebrafish, N = 6, oneway ANOVA with Tukey's post-hoc test, ∗p < 0.05, ∗∗p < 0.01, ∗∗∗p < 0.001, ∗∗∗∗p < 0.0001. (b) IBR calculated the star plot of antioxidant enzyme activity in the brain tissue of zebrafish larvae injected with empty vector mutants, overexpressing *slc7a11* mutants, and overexpressing *foxo1a* mutants with *slc7a11* knockdown. MT represents *foxo1a* mutant zebrafish.

Next, to investigate whether there is a direct relationship between foxola and the oxidative stress pathway, we used the DeepPBS tool [37] to individually examine the promoter regions of these downstream affected genes (Fig. 6A). Analysis revealed that the promoters of *slc7a11* contain multiple conserved *foxo1a* response element sequences (Fig. 6B). Interestingly, prior RT-qPCR results showed that the transcription levels of *slc7a11* was most significantly regulated by *foxo1a*. This suggests that the transcription factor *foxo1a* is likely to exert its antioxidant protective function by directly activating the transcription of *slc7a11*.

To further verify the regulatory relationship between *foxo1a* and *slc7a11*, we conducted a dual-luciferase reporter assay to examine the transcriptional regulation of the *slc7a11* gene by *foxo1a.* The results showed that the relative luciferase activity increased significantly in the presence of both the expression plasmid pC3.1-*foxo1a* and the reporter plasmid pGL-*slc7a11* (Fig. 6B).

To determine the binding location of the *foxo1a* transcription factor on the *slc7a11* gene promoter, we performed a fragment deletion experiment. The results indicated a significant difference between the first and second binding sites, suggesting that the transcriptional regulatory site of the *foxo1a* transcription factor on the *slc7a11* gene promoter is located between −1020 and −1014 (Fig. 6B).

To confirm that *slc7a11* is indeed a key downstream target through which the *foxo1a* gene exerts its antioxidant protective function, we exogenously supplemented *slc7a11* overexpression plasmids in *foxo1a* gene-deficient zebrafish for rescue validation. We found that compared to *foxo1a* gene-deficient zebrafish injected with an empty vector, exogenous supplementation of *slc7a11* effectively restored the abnormal myelin ultrastructure in the brain tissue of *foxo1a* gene-deficient zebrafish (Fig. 6C). It also enhanced the fluorescence intensity of the oligodendrocyte marker Olig2 (Fig. 6D) and restored the expression of oligodendrocyte development-related marker genes such as *mbp, plpa, gfap* and *s100b* (Fig. 6F). Additionally, we found that exogenous supplementation of *slc7a11* successfully reduced ROS levels in the brain tissue of *foxo1a* gene-deficient zebrafish (Fig. 6E) and enhanced the mRNA expression of various antioxidant genes (*sod, ho1, gpx4, nrf2*) (Fig. 6H). Correspondingly, the activity of multiple antioxidant enzymes (SOD, CAT, GSH) was also enhanced (Fig. 6G).

To verify that *slc7a11* is a key downstream gene of *foxo1a* in exerting antioxidative protective functions in the central nervous system, it is necessary to conduct a combined rescue experiment of *foxo1a* overexpression and *slc7a11* knockdown in *foxo1a* gene-deficient zebrafish. First, we validated the efficiency of Si*-slc7a11* in wild-type zebrafish embryos by injecting 50 nm of Si-*slc7a11* exogenously into one-cell stage embryos of wild-type zebrafish. The results showed that the expression of the *slc7a11* gene in wild-type zebrafish embryos was significantly downregulated 4 days post-injection (Fig. S2). On this basis, we supplemented *foxo1a* overexpression plasmids exogenously in embryos of *foxo1a* gene-deficient zebrafish while simultaneously injecting 50 nm of Si-*slc7a11*. We found that exogenous supplementation with *foxo1a* overexpression plasmids, along with the simultaneous injection of 50 nm Si-*slc7a11*, significantly weakened the rescue effect of the *foxo1a* overexpression plasmid on the abnormal phenotype of *foxo1a* gene-deficient zebrafish (Fig. 6F, 6H, 6G). This indicates that *slc7a11* is indeed a key downstream gene for *foxo1a* in exerting antioxidative protective functions in the central nervous system.

10. Overexpression of foxo1a effectively alleviates oligodendrocyte oxidative damage induced by CuSO_4_.

**Fig. 7:**
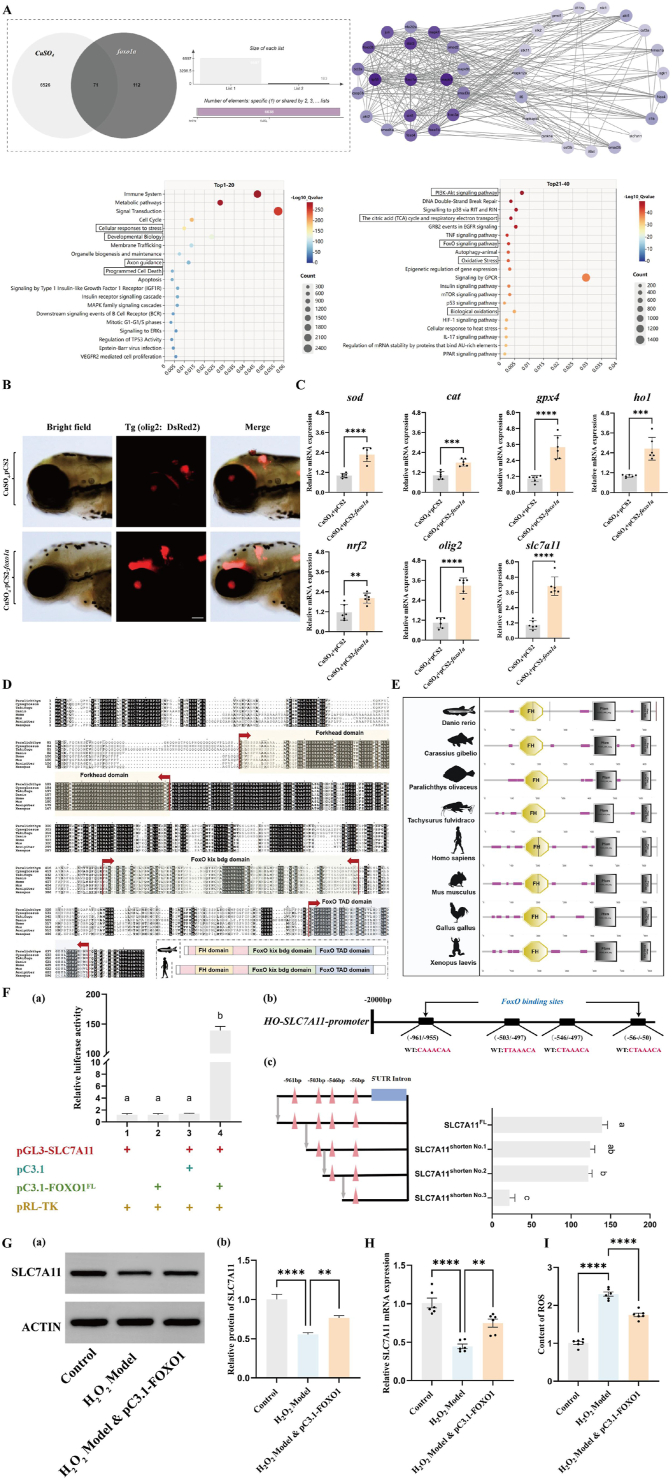
Application of *foxo1a* in the CuSO4-induced demyelination model. A: Network molecular analysis results. (a) Venn diagram showing the number of common targets between *foxo1a* and CuSO_4._ (b) KEGG and GO enrichment analysis of common targets. B: *olig2* fluorescence expression in the brains of zebrafish larvae from the control group and the overexpressed *foxo1a* group under CuSO_4_ treatment, scale bar = 100 μm C: mRNA expression levels of *sod, cat, ho1, gpx4, nrf2, olig2,* and *slc7a11* in the brain tissue of zebrafish larvae from the control group and the overexpressed *foxo1a* group under CuSO_4_ treatment, N = 6, *t*-test, ∗p < 0.05, ∗∗p < 0.01, ∗∗∗p < 0.001, ∗∗∗∗p < 0.0001. D: Protein sequence alignment of *foxo1a.* E: Structural analysis of representative proteins from the *foxo1a* family. F: (a) pGL3 and pC3.1 represent the circular pGL3-basic plasmid and circular pcDNA3.1 (+) plasmid, respectively. The SLC7A11 gene promoter sequence was homologously recombined with the linearized pGL plasmid to construct the reporter plasmid pGL-SLC7A11. The full-length CDS sequence of FOXO1 was homologously recombined with the linearized pcDNA3.1 (+) plasmid to construct the expression plasmid pC3.1-FOXO1. The pRL-TK plasmid expressing rluc was co-transfected in each group to characterize transfection efficiency. The Arabic numerals near the x-axis indicate different treatment groups. Different letters indicate significant differences (P < 0.05). (b) Analysis diagram of the FOXO1-specific binding site on the SLC7A11 gene promoter. (c) Fragment deletion experiment of the transcriptional regulation of the SLC7A11 gene by transcription factor FOXO1. The pink triangles (△) represent FOXO1 transcription factor binding sites predicted by DeepPBS software. The four black horizontal lines represent the lengths of the promoter sequences in the reporter plasmids. They were obtained by homologous recombination of pGL3 basic plasmid fragments with SLC7A11 gene promoter sequences of different lengths. Different letters indicate significant differences (P < 0.05). G: (a) In human oligodendrocytes, the expression of SLC7A11 protein in the control group, the H_2_O_2_ model group, and the H_2_O_2_ model group with overexpressed FOXO1 was evaluated (b) the relative quantification of the SLC7A11 protein was performed using normalization, oneway ANOVA with Tukey's post-hoc test, ∗p < 0.05, ∗∗p < 0.01, ∗∗∗p < 0.001, ∗∗∗∗p < 0.0001. H: In human oligodendrocytes, the relative expression of SLC7A11 mRNA in the control group, H_2_O_2_ model group, and H_2_O_2_ model group overexpressing FOXO1, oneway ANOVA with Tukey's post-hoc test, N = 6 ∗ p < 0.05, ∗∗p < 0.01, ∗∗∗p < 0.001, ∗∗∗∗p < 0.0001. I: In human oligodendrocytes, the ROS levels in the control group, the H_2_O_2_ model group, and the H_2_O_2_ model group overexpressing FOXO1, oneway ANOVA with Tukey's post-hoc test, N = 6∗ p < 0.05, ∗∗p < 0.01, ∗∗∗p < 0.001, ∗∗∗∗p < 0.0001.

To evaluate the specificity and effectiveness of the *foxo1a* target in myelin antioxidant protection, we adopted the widely recognized CuSO_4_ myelin model. First, using computer-aided drug screening strategies through enrichment analysis and clustering analysis, we found that the CuSO_4_-induced myelin injury model is associated with pathways such as cellular stress response, biological development, axon guidance, programmed cell death, the TCA cycle, and oxidative stress (Fig. 7A). Subsequently, we transfected-labeled transgenic zebrafish embryos through microinjection and exposed them to 5 mmol CuSO_4_ for 4 days. We observed that the oligodendrocytes in the brains of transgenic zebrafish larvae expressing pCS2-*foxo1a* were less damaged by CuSO_4_ compared to those carrying an empty vector. (Fig. 7B). Meanwhile, RT-PCR results showed that transgenic zebrafish larvae expressing pCS2-*foxo1a* exhibited lower oxidative stress levels in the brain, and the expression of slc7a11 increased. (Fig. 7C), further demonstrating the critical role of *foxo1a-slc7a11* in myelin antioxidant protection.

11. Genetic and physiological significance of foxo1a in adaptive evolution

To investigate whether the antioxidant protective role of *foxo1a* in the myelin sheath and nervous system of zebrafish, as discovered in our previous research, represents a molecular convergent evolution shared by jawed vertebrates, this study conducted a comparative analysis of the amino acid sequences and protein domains of *foxo1a* across 14 closely and distantly related species. Multiple sequence alignment revealed that the CDS region of *foxo1a* in jawed vertebrates primarily comprises three structural modules: one Forkhead domain, one FoxO kix bdg domain, and one FoxO TAD domain. Among these, the Forkhead domain was found to be highly conserved in several representative jawed vertebrates (Fig. 7D). Further analysis of the arrangement and combination of *foxo1a* protein domains showed that the arrangement and composition of the functional domains of *foxo1a* are highly conserved among eight jawed vertebrates (Fig. 7E). To further demonstrate that the *foxo1a-slc7a11* axis is a convergent molecular mechanism in jawed vertebrates, we conducted dual fluorescence verification in human 293T cells. We found that in human cells, when the expression plasmid pc3.1-FOXO1 and the reporter plasmid pGL-SLC7A11 were present simultaneously, the relative luciferase activity value was significantly higher than that of other control groups. To determine the binding site of the FOXO1 transcription factor on the SLC7A11 gene promoter, we first used the DeepPBS tool to analyze and identify potential binding sites in the SLC7A11 promoter region (Fig. 7F). Based on these findings, we conducted a fragmentation deletion experiment, which revealed a significant difference between binding sites −546 and −497, indicating that the FOXO1-SLC7A11 axis is also an evolutionarily conserved molecular mechanism in human cells (Fig. 7F). In addition, we conducted functional validation in human oligodendrocyte cells (MO3.13). It has been reported that 500 μmol/L of H_2_O_2_ is used to construct an effective oxidative damage model of MO3.13. Since oxidative damage mediates changes in intracellular antioxidant genes and ROS content, we examined the expression of the SlC7A11 and the content of ROS in cells of each group through RT-qPCR, Western blotting and ELISA. The results showed that compared to the control group, the expression level of mRNA SLC7A11 gene in the H_2_O_2_ model group MO3.13 cells was significantly reduced (Fig. 7H), and SLC7A11 protein is relatively reduced (Fig. 7G). The content of ROS was significantly increased (Fig. 7I). On this basis, in the H_2_O_2_ model group MO3.13 cells transfected with the FOXO1 overexpression plasmid, the expression level of mRNA SLC7A11 gene was restored (Fig. 7H), and SLC7A11 protein is relatively restored (Fig. 7G). The ROS content was also significantly reduced (Fig. 7I). This indicates that FOXO1-SLC7A11 is also a key antioxidant signaling pathway in human oligodendrocyte cells. Referring to our previous research, the *foxo1a* gene first appeared in the genome of jawed vertebrates and was preserved throughout subsequent evolution, strongly suggesting that *foxo1a* is indeed an adaptive genetic basis for antioxidant protection in the nervous system, including myelin, in jawed vertebrates.

## 3 Discussion

We have revealed for the first time that *foxo1a* is a key regulatory factor in antioxidant defense and myelin integrity within the central nervous system. In this study, we found that the entry of *foxo1a* into the genome of jawed vertebrates coincided remarkably with the appearance of myelin, suggesting that this gene may play an important role in helping jawed vertebrates achieve complex neural signal conduction. Subsequently, we discovered that *foxo1a* is directly localized in oligodendrocytes and myelin during early embryonic development and plays a crucial role in maintaining myelin development and resisting oxidative stress. Mechanistically, the interaction between *foxo1a* and *slc7a11* reveals a key molecular pathway for antioxidant protection and mitigation of iron toxicity in the central nervous system. In terms of application, the overexpression of *foxo1a* successfully rescued CuSO_4_-induced neural oxidative damage, further enhancing the potential application value of this target. These findings not only elucidate the neuroprotective mechanisms of *foxo1a* but also provide a framework for understanding how antioxidant defenses evolved to support the increased complexity of the vertebrate nervous system.

Divergence time estimation is a central focus in contemporary macroevolutionary studies, employing the extent of genetic sequence divergence alongside molecular clocks to estimate divergence times between lineages and to determine the timing of other nodes on phylogenetic trees. This approach facilitates the inference of the origins and divergence times of related groups [38]. Our investigation revealed that the *foxo1a* gene was integrated into vertebrate genomes approximately 430 million years ago, coinciding with the transition from the Ordovician to Silurian periods and the divergence of jawed vertebrates from primitive agnathans [8]. The turnover of vertebrate species during the Ordovician and Silurian periods was marked by mass extinctions followed by the reoccupation of ecological niches, with the emergence of numerous vacant niches driving the rapid diversification of jawed vertebrates [39]. Notably, following its incorporation into the genomes of jawed vertebrates, the *foxo1a* gene underwent a highly conserved expansion, as supported by interspecies synteny analyses. Gene family expansion is a fundamental aspect of biological evolution, illustrating how organisms enhance genetic diversity and adaptability through gene duplication and variation in response to environmental pressures and survival challenges [40]. A significant distinction between the evolution of agnathans and jawed vertebrates is the development of myelin [41]. Unlike the multilayered glial sheaths found in agnathans, the evolution of myelin in jawed vertebrates provided the advantage of rapid signal transmission while maintaining relatively small axon diameters [42,43]. To specifically examine whether the expansion of the *foxo1a* gene is associated with myelin, we conducted a reanalysis of publicly available single-cell RNA-sequencing datasets from zebrafish brain samples, a model organism for jawed vertebrates. Our findings indicate that *foxo1a* is highly expressed in cell clusters related to myelin and oligodendrocytes. The normal differentiation of oligodendrocytes is crucial for the myelination of axons in the central nervous system. From a developmental perspective, the period during which oligodendrocytes in the brain wrap around axons to form myelin coincides with the critical period when *olig2* exerts its regulatory function in oligodendrocytes. This suggests that the transcription factor encoded by the *olig2* gene plays an important regulatory role in the development and differentiation of oligodendrocytes [44]. In this study, we found that the expression of *olig2* is significantly reduced at both the fluorescence intensity and mRNA levels following the deletion of the *foxo1a* gene. This indicates that there may be abnormalities in the development or differentiation of oligodendrocytes. Considering that it is a key regulatory factor in oligodendrocyte differentiation and myelination, we further detected changes in the myelin structure. Specifically, in the brains of *foxo1a* gene-deficient zebrafish, myelin boundaries appeared blurred, compact myelin was absent on axons, and myelin structures were either loose or thinner. Furthermore, the mRNA levels of myelin basic protein (*mbp*), a marker gene specific to the myelin of jawed vertebrates [[45], [46], [47]], were significantly reduced. This study provides the first direct evidence linking the *foxo1a* gene with oligodendrocyte development, differentiation, and myelination. Nevertheless, while we have established an association between *foxo1a* and myelin, elucidating the precise molecular mechanisms by which *foxo1a* influences myelin and oligodendrocytes remains a substantial challenge.

During neural development, oligodendrocytes are primarily responsible for myelin formation; however, they also possess the capacity to differentiate into astrocytes [34]. Consequently, we explored whether astrocytes were similarly impacted in the brains of zebrafish deficient in *foxo1a* gene. Consistent with our hypothesis, astrocyte markers, such as *gfap*, were significantly diminished in the brains of *foxo1a* gene-deficient zebrafish, alongside a notable reduction in the activity of glutamine synthetase. Previous research has demonstrated that glutamine synthetase in astrocytes facilitates the conversion of glutamate to glutamine, which is subsequently supplied back to neurons [48]. This conversion is crucial for the recycling of glutamate. Our findings suggest that the brains of *foxo1a* gene-deficient zebrafish may undergo transient disruptions in excitatory-inhibitory neural circuits. It is well-established that the peak of glutamate binding to NMDA receptors occurs during early adolescence, and even minor alterations in glutamate levels during this critical period can result in frequent psychiatric disturbances in juveniles [49,50]. The light-dark box (LDB) test is a well-established method for assessing anxiety and depression-like behaviors [51]. In our study, zebrafish deficient in *foxo1a* gene demonstrated significant freezing behavior when exposed to light stimulation a response characterized by the cessation of movement, heightened mental tension, and fear which serves as a critical indicator of atypical psychiatric behavior in animals. While these findings do not directly clarify the molecular role of *foxo1a* in the processes of oligodendrocyte differentiation and myelination, they offer preliminary evidence suggesting abnormalities in oligodendrocytes and myelin.

Studies on gene expression regulation are crucial for elucidating the mechanisms underlying phenotypic traits in various species [52]. For instance, variations in foraging and swimming performance among different ecotypes of Arctic char are influenced by alternative splicing and differential gene expression [53]. The freshwater fish species *Melanotaenia duboulayi* demonstrates significant plasticity in response to thermal stress through alterations in gene expression levels [54]. In light of this, our initial investigation focused on the relationship between the *foxo1a* gene and molecules associated with oligodendrocytes and myelin. Employing the general DeepPBS model, as published in *Nature Methods* [37], we conducted an analysis of the DNA-binding specificity of the transcription factor *foxo1a* in relation to the promoter regions (2000 bp upstream) of several differentially expressed genes. Regrettably, our study did not identify highly specific binding sequences. Despite observing aberrant transcriptional regulation in several genes, such as *olig2* and *mbp,* we did not obtain direct evidence establishing a connection between *foxo1a* and the transcriptional regulation of these genes. Consequently, we propose that the developmental abnormalities observed in oligodendrocytes and myelin in *foxo1a* gene-deficient zebrafish may represent secondary effects, necessitating further investigation to elucidate this matter.

It is noteworthy that neuronal cells exhibit exceptionally high energy demands compared to other cell types [55]. Under normal physiological conditions, neurons predominantly generate ATP via mitochondrial oxidative phosphorylation, a process that unavoidably results in the production of ROS due to electron leakage from the mitochondrial respiratory chain [[56], [57], [58]], and oligodendrocyte precursor cells are more sensitive to oxidative stress than mature oligodendrocytes [59]. In jawed vertebrates, the evolution of more complex neural structures, including myelination, has conferred not only enhanced capabilities for rapid and efficient neural signal transmission [43] but also an increased reliance on antioxidant defense mechanisms [11]. Previous research has demonstrated that the FoxO protein family plays a significant role in the regulation of cellular oxidative stress [60]. Consequently, we hypothesize that the developmental abnormalities in oligodendrocytes and myelin observed in *foxo1a* gene-deficient zebrafish may be attributable to compromised antioxidant defenses. The activities of various antioxidant enzymes are frequently employed as indicators of the intensity of oxidative stress defense. Among these enzymes, SOD is recognized as the primary defense mechanism against superoxide radicals, facilitating their conversion into hydrogen peroxide [61]. In our investigation, SOD activity was markedly diminished in zebrafish deficient in *foxo1a* gene, which may result in the accumulation of intracellular ROS and a disruption of the antioxidant defense system. In addition, the activity of CAT, which is responsible for detoxifying hydrogen peroxide (H_2_O_2_) to prevent lipid peroxidation of cell membranes, and the intracellular antioxidant molecule GSH, also show significantly reduced activity in foxo1a gene-deficient zebrafish [62,63]. The decrease in SOD and CAT activity may lead to the dual accumulation of O_2_^−^ and H_2_O_2_, forming a vicious cycle, while the decrease in GSH activity may further exacerbate cellular oxidative damage [[64], [65], [66]]. In addition, we also found that the ROS level in the brain of zebrafish with *foxo1a* deficiency was significantly increased. It has been reported that the production of ROS can rapidly attack macromolecules such as phospholipids, membrane receptors, and enzyme-related polyunsaturated fatty acids on biological membranes, causing lipid peroxidation and forming lipid peroxides [67,68]. In this study, the significant increase in the level of lipid peroxidation product MDA observed in the brains of zebrafish with foxo1a gene deletion is likely primarily due to lipid peroxidative damage caused by excessive ROS. In conclusion, these results strongly suggest that the *foxo1a* gene is crucial for antioxidant protection in the zebrafish brain.

The impact of oxidative stress on neural damage is predominantly exhibited through both direct and indirect harm to neural cells [69]. Directly, oxidative stress can lead to lipid peroxidation of neural cell membranes, resulting in increased permeability, disruption of cellular function, and induction of apoptosis [70]. In this investigation, flow cytometry analysis demonstrated that deficiency in *foxo1a* gene indeed precipitated brain cell death, underscoring the essential role of *foxo1a* in cell survival. Nonetheless, further evidence is required to ascertain whether this is directly attributable to compromised antioxidant defenses. Importantly, the generation of lipid peroxides is a characteristic feature of ferroptosis, a regulated form of cell death [71]. Based on our observations of elevated malondialdehyde (MDA) levels in the brain, we propose that cell death in *foxo1a* gene-deficient zebrafish may involve ferroptosis. The main mechanism of ferroptosis is that under the action of divalent iron or lipoxygenase, highly expressed unsaturated fatty acids on the cell membrane are catalyzed, leading to lipid peroxidation, which in turn induces cell death [[71], [72], [73]]. In the flow cytometry experiment, we happen to detect cell death through Annexin V binding to phosphatidylserine on the outer cell membrane. Combined with the significant increase in iron ion levels in the brains of mutants, it is not difficult to ascertain that the deficiency of *foxo1a* gene induces brain cell death in a manner that is at least partially related to ferroptosis. This is largely because the harmful oxidative stress levels within the cells provide a prerequisite for the occurrence of ferroptosis.

Following the elucidation of the neuroprotective antioxidant function of *foxo1a*, our research focus transitioned to exploring the molecular regulatory interactions between *foxo1a* and genes implicated in antioxidant defense and ferroptosis. Utilizing the DeepPBS model, we conducted an analysis of the promoter regions (spanning 2000 base pairs upstream) of genes associated with these processes. Notably, we have identified several previously unreported specific *foxo1a* binding sites in the promoter of *slc7a11*. Furthermore, dual-luciferase reporter assays substantiated that *foxo1a* directly modulates the transcriptional activity of *slc7a11*, we also validated and confirmed that after the loss of the *foxo1a* gene, the transcription and protein levels of *slc7a11* and its downstream genes were significantly reduced, thereby unveiling a novel mechanism of action. Under normal physiological conditions, the light chain subunit of *slc7a11* is crucial for the synthesis of reduced glutathione [74], which is essential for maintaining intracellular and extracellular redox balance and regulating intercellular signaling [75]. The regulatory influence of *foxo1a* on *hamp1* has been previously documented [76]. Research has indicated that reduced expression of *hamp1* leads to diminished production of functional hepcidin, resulting in increased iron absorption [77].The previously observed increase in Fe^2+^ concentrations in the brains of mutant zebrafish is likely attributable to the transcriptional suppression of *hamp1*, as evidenced by reduced *hamp1* mRNA expression levels. These findings collectively suggest that, in the deficiency of *foxo1a*, the dysregulation of peroxide and iron ion homeostasis intensifies Fenton reactions, resulting in the excessive accumulation of lipid peroxides and the induction of ferroptosis, which ultimately impairs neural cell differentiation and development.

The potential for off-target effects resulting from gene-editing technologies, which may lead to false positives, cannot be disregarded [78]. To this end, we further conducted overexpression experiments to verify whether the abnormal phenotypes such as oxidative stress damage, iron overload, oligodendrocyte abnormalities, and anxiety-like behavior in the brains of *foxo1a* gene-deficient zebrafish larvae could be successfully rescued. First, the effectiveness of *foxo1a* overexpression in vivo was verified. Compared to *foxo1a* gene-deficient zebrafish larvae injected with an empty vector, those injected with the pCS2-*foxo1a* plasmid showed a significant increase in *foxo1a* mRNA levels in the brain. Additionally, the levels of ROS and Fe^2+^ in the telencephalon were significantly downregulated, and the fluorescence intensity of olig2 was restored. Furthermore, the transcription levels of *hamp1, slc7a11,* and its downstream genes were also restored in the brains of *foxo1a* gene-deficient zebrafish larvae injected with the pCS2-*foxo1a* plasmid. To elucidate the regulatory relationship between *foxo1a* and *slc7a11*, we overexpressed *slc7a11* in *foxo1a* gene-deficient zebrafish larvae to verify whether the oxidative stress damage caused by its deficiency was due to the transcriptional repression of *slc7a11*. We found that overexpressing *slc7a11* not only activated the mRNA expression of previously inhibited downstream genes encoding antioxidant enzymes but also restored the fluorescence intensity of olig2 and normalized the ROS levels in the brain. Moreover, transmission electron microscopy results showed that the previously abnormal myelin ultrastructure in the brains of *foxo1a* gene-deficient zebrafish overexpressing *slc7a11* was also repaired. To better emphasize that *slc7a11* is a key downstream factor in the antioxidative function of the *foxo1a* gene in the nervous system, we additionally conducted a combined rescue experiment of *slc7a11* knockdown and *foxo1a* overexpression, successfully demonstrating that the transcription factor *foxo1a* plays a crucial role in the antioxidative defense of zebrafish neural cells by directly regulating the activity of the *slc7a11* promoter.

In order to further investigate the effective application of *foxo1a* targets in the antioxidant protection of myelin in jawed vertebrates, we utilized an animal model widely recognized for inducing myelin damage to verify the antioxidant protective effects of *foxo1a*. It is worth mentioning that network pharmacology analysis found that the biological processes such as oxidative stress response and ferroptosis regulated by the *foxo1a* gene, which were confirmed in this study, are also key targets in the CuSO_4_-induced myelin injury model. Correspondingly, 5 mmol of CuSO_4_ significantly reduced the fluorescence intensity of the oligodendrocyte marker olig2 in the central nervous system of wild-type zebrafish and promoted the production of ROS. On this basis, exogenous supplementation with *foxo1a* can significantly alleviate oxidative damage in the central nervous system caused by CuSO_4_. This suggests that the *foxo1a* target has good application prospects in antioxidant protection in the central nervous system.

The evolution of myelin in jawed vertebrates is an important biological process that significantly increases the speed of nerve impulse conduction along axons [33]. The emergence of this feature has enabled jawed vertebrates to achieve significant evolutionary advantages in terms of nervous system function. However, myelin is not just an insulator; it also participates in the energy metabolism process of neurons. It has been reported that oligodendrocytes and Schwann cells in myelin play important roles in preventing electrical coupling and providing metabolic support, but at the same time, they also introduce higher oxidative stress [[79], [80], [81]]. This is because the proliferation and differentiation of oligodendrocytes primarily rely on oxidative phosphorylation to provide energy [82,83]. The process of oxidative phosphorylation can increase the production of ROS, which may cause oxidative damage to cells [84,85]. However, jawed vertebrates seem not to be affected by stronger oxidative stress while maintaining efficient signal transduction. Based on the findings of this study, we hypothesize that the *foxo1a-slc7a11* axis found in the model organism zebrafish may serve as a common neuroprotective antioxidant mechanism in jawed vertebrates. In the study of molecular evolution, methods such as multiple sequence alignment, functional domain rearrangement, and chromosome synteny analysis are effective approaches to explore whether gene functions can be transformed through homology [86,87]. Based on this, we conducted a comparative analysis of the amino acid sequences of the *foxo1a* gene in different species and found that the amino acid sequences of the *foxo1a* gene are highly conserved among various jawed vertebrates. Amino acid sequences usually determine the structure and function of proteins, and the conservation of these sequences supports the potential for the *foxo1a* gene function to be conserved among jawed vertebrates. Furthermore, the arrangement and composition of functional domains are also found to be a core driving force in the functional complexity and diversification of biological evolution [88,89]. In this study, we found that the *foxo1a* gene in various jawed vertebrates exhibits a high degree of consistency in both the composition and arrangement of its functional domains. Although the aforementioned analysis supports the possibility that the *foxo1a-slc7a11* axis may serve as a convergent molecular mechanism and function in the evolution of antioxidant protection in jawed vertebrates, more specific experimental validation would be beneficial for this hypothesis. To this end, we further verified the conservation of the *foxo1a-slc7a11* axis in human cells, successfully demonstrating that this axis functions as a convergent molecular strategy for antioxidant protection in the central nervous system of jawed vertebrates.

In conclusion, this study elucidates the pivotal protective function of the transcription factor *foxo1a* in mitigating oxidative damage in myelin by directly regulating the transcription of *slc7a11*. The emergence of myelin in jawed vertebrates was instrumental in the evolution of complex nervous systems, yet it concurrently heightened their reliance on mechanisms for oxidative stress protection. The expansion of *foxo1a* in these organisms provided a robust genetic foundation for their rapid adaptive evolution and environmental adaptation. This genetic integration of endogenous *foxo1a* into the genome of jawed vertebrates is closely associated with the evolutionary development of myelin. These findings not only enhance our comprehension of the distinctions and interconnections between the adaptive evolution of jawless and jawed vertebrates but also furnish essential data and theoretical support for investigating the evolutionary characteristics of neural defense mechanisms.

## 4 Materials and methods

### 4.1 Animals and housing

Adult AB strain zebrafish (Danio rerio), *foxo1a*^ihb390/+(AB)^, and Tg (olig2: DsRed2) used in this study were obtained from the China Zebrafish Resource Center (CZRC). The fish were placed in an automated recirculating water system (Haisheng, Shanghai, China) under a natural light cycle of approximately 14 h of light and 10 h of darkness, with the water temperature maintained at 28 ± 0.5 °C. Zebrafish were fed live Artemia nauplii twice daily. To ensure optimal water quality, uneaten food was removed from the tank 30 min after feeding. The experimental protocol was approved by the Animal Ethics Committee of Shanghai Ocean University and complied with the “Guidelines for the Ethical Treatment of Laboratory Animals” issued by the Ministry of Science and Technology of China.

### 4.2 Phylogenetic analysis of gene families

The amino acid sequences of *foxo1a* proteins from multiple species were selected for constructing a phylogenetic tree, with all sequences obtained from the NCBI database (https://www.ncbi.nlm.nih.gov/). The phylogenetic tree was constructed using the Neighbor-Joining method (Saitou and Nei, 1987) in MEGA 11.0 (Tamura et al., 2021) software. The phylogenetic tree was refined using the online tools iTOL (https://itol.embl.de/) and timetree (http://www.timetree.org/home).

### 4.3 Analysis of gene contraction and expansion, chromosome localization, and species synteny

Gene family contraction and expansion were performed using the OrthoVenn3 platform (https://orthovenn3.bioinfotoolkits.net/). Chromosome localization was determined by downloading zebrafish genome sequences and annotation files from the NCBI database (https://www.ncbi.nlm.nih.gov/), visualizing the location of FoxO gene families on chromosomes using TBtools, and examining gene structure and conserved domains using SMART (http://smart.embl-heidelberg.de/). Synteny analysis of FoxO gene families in zebrafish and lamprey was conducted using TBtools.

### 4.4 Single-cell analysis

We used the Daniocell website (https://daniocell.nichd.nih.gov/) for labeling and visualizing clusters with high expression of *foxo1a* and oligodendrocyte genesis genes. Use Adobe AI software to encircle the oligodendrocyte population we focus on with black dashed lines for emphasis.

### 4.5 Pathway enrichment analysis

Retrieve CuSO_4_ target genes through the GeneCards database (https://www.genecards.org/), and construct a protein-protein interaction network for the foxo1a gene using the STRING database (https://string-db.org/) to screen for its functionally related genes. Use the jvenn online tool (https://jvenn.toulouse.inra.fr/) to draw a Venn diagram to analyze the overlap between CuSO_4_ target genes and foxo1a interacting genes. The overlapping genes mentioned above were analyzed and optimized for protein interaction networks using Cytoscape 3.10.1 software. Submit the above overlapping target genes to the Metascape platform (https://metascape.org/) for KEGG pathway and GO enrichment analysis and refined the results using the Chiplot website. Set the species background to zebrafish to explore the synergistic mechanism of the two in zebrafish.

### 4.6 Establishment of mutant fish lines

The *foxo1a* heterozygous mutant zebrafish (CZRC catalog number: CZ1342) purchased from the China Zebrafish Resource Center were inbred for generations and raised to 90 days of age for genotyping. First, tail clipping was performed to extract DNA, followed by preliminary genotype analysis using high-resolution melting curves, and then sent to Shanghai Sangon Biotech for final validation through sequencing.

### 4.7 Histological analysis

For whole-mount in situ hybridization analysis, the open reading frames of *olig2* and *mbp* genes were cloned into the pGEM-T easy vector (Promega), then linearized to synthesize riboprobes. Sense and antisense riboprobes were synthesized for each gene. Sense riboprobes were used as negative controls to estimate staining reaction time and determine the specificity of antisense riboprobes. Riboprobes were generated by in vitro transcription with T7 RNA polymerase and labeled with Digoxigenin-11-UTP (Roche), then purified using Illustra Microspin G-25 columns (GE Healthcare) according to the manufacturer's instructions. Zebrafish embryos were fixed in 4 % paraformaldehyde and permeabilized in 100 % methanol at −20 °C for at least 10 h. Subsequently, embryos were rehydrated and digested with Proteinase K. Embryos were then hybridized with riboprobes at 68 °C for at least 15 h. Unbound and excess probes were removed by washing three times in 50 % formamide, followed by washing three times in 2 × SSC 0.1 % Tween-20 and 0.2 × SSC 0.1 % Tween-20. After three washes with PBST (PBS plus 0.1 % Tween-20), embryos were blocked with 5 % sheep serum at room temperature for 1 h, then incubated overnight at 4 °C with sheep anti-Digoxigenin alkaline phosphatase (1:2000; Roche) and washed gently six times in PBST for 24 h. Color development was performed in the dark at room temperature using NBT/BCIP substrate (Roche).

### 4.8 Western blotting

Protein samples were extracted using RIPA buffer supplemented with protease inhibitors. Protein concentrations were determined by the BCA assay. Samples were denatured in loading buffer at 100 °C for 15 min. Protein separation was performed by SDS-PAGE, followed by transfer onto activated PVDF membranes (0.45 μm) at 350 mA for 30 min. Membranes were blocked with 5 % milk in TBST at room temperature for 30 min, incubated overnight at 4 °C with primary antibodies (ACTIN, Beyotime, AF0003, 1:1000; HO-1, Beyotime, AF1333, 1:1000; GPX4, Beyotime, AF7020, 1:1000; SLC7A11, Beyotime, AF7992, 1:1000), and subsequently incubated with HRP-conjugated secondary antibodies for 30 min at room temperature. Signals were visualized using enhanced chemiluminescence (ECL) and captured by a chemiluminescence imaging system. Image quantification and analysis were conducted with AIWBwell software, calculating relative protein expression from integrated density values adjusted by internal control.

### 4.9 Establishment of reactive oxygen species (ROS) model in Mo3.13 cells

Inoculate MO3.13 at the same growth stage into multiple 24-well plates. Based on previous reports, select 500 μmol L^−1^ H_2_O_2_ as the optimal concentration for modeling. Use a random number table to randomly divide MO3.13 into three groups: control group, H_2_O_2_ model group, and H_2_O_2_ & pC3.1-FOXO1 group. After incubating for 24 h in a 37 °C CO_2_ incubator, collect the aforementioned MO3.13 for subsequent related experiments.

### 4.10 Knockdown of *slc7a11* gene in zebrafish by morpholino injection

To achieve transient knockdown of the zebrafish *slc7a11* gene, antisense morpholino oligonucleotides (MO) targeting the translation initiation site of *slc7a11* were designed and synthesized (Gene Tools, LLC, Philomath, OR, USA). The sequence of *slc7a11* MO was as follows: 5′-CCTGTATCCGATCACACTA-3′ (replace with actual sequence). A standard control morpholino (Ctrl-MO) provided by Gene Tools was used as a specificity control. Morpholino oligonucleotides were diluted to a working concentration of 0.5 mM with sterile nuclease-free water containing 0.05 % phenol red. Zebrafish embryos at the one to two-cell stage were microinjected with approximately 2–4 ng *slc7a11* MO or an equivalent dose of Ctrl-MO, using a pneumatic microinjector. After injection, embryos were incubated at 28.5 °C in E3 medium, and morpholino efficacy was confirmed by reverse transcription-quantitative PCR (RT-qPCR) analysis at 96 hpf.

### 4.11 Detection of ROS content

ROS localization was detected using the H2DCFDA probe, which emits green fluorescence upon excitation (EX/Em: 488 nm/525 nm), directly reflecting the degree of oxidative radical changes based on fluorescence intensity. The ROS probe stock solution was 10 mM, and the working concentration was diluted to 5 μM ROS detection solution with PBS. Zebrafish larvae were washed twice with PBS, placed into 1.5 ml brown EP tubes, and immediately incubated with the probe at 28 °C in the dark for 15 min. After incubation, the ROS detection solution was quickly aspirated, and the larvae were washed three times with 1 ml PBS. Larvae were gently transferred to methylcellulose using a Pasteur pipette, and embryos were positioned using an egg picker. Fluorescence microscopy parameters were fixed, and all embryos were imaged using identical imaging settings to detect fluorescence conditions and take photographs.

In the cell experiments, the changes in ROS levels among the groups were detected. The treated cells were immersed in a water bath at 95 °C for 40 min, then removed and rinsed with cold water. After cooling, the cells were centrifuged at 4000 rpm for 10 min. The tissue homogenate was mixed with 2,7-dichlorodihydrofluorescein diacetate (DCF DA) at 37 °C for 15 min, then centrifuged at 10000 rpm for 15 min. The supernatant was discarded, and the pellet was resuspended in PBS and incubated at 37 °C for 60 min. The ROS levels were subsequently measured using a spectrophotometer.

### 4.12 Enzyme activity assay

Zebrafish larvae from each group were precisely weighed, and homogenization fluid was added at a weight-to-volume ratio of 1:9, with 0.86 % or 0.9 % saline recommended. The homogenate was prepared at 10 % using mechanical homogenization under an ice-water bath. Subsequently, the homogenate was centrifuged at 2500–3000 rpm for 10 min, and the supernatant was collected for further analysis. SOD, CAT, GSH, and MDA detection kits were purchased from Nanjing Jiancheng. Experimental procedures should strictly follow the protocols provided in the kit instructions.

### 4.13 IBR analysis

The Hiplot website (https://hiplot.com.cn/home/index.html) was used to calculate the star plot area or integrated index of enzyme activity data from different groups to obtain IBR values. These values were used to assess the integrated biological response of zebrafish larvae across different groups. Normalized data were then plotted as star plots to visually present the responses of different biomarkers.

### 4.14 Iron ion content measurement

Zebrafish larvae were washed twice with HBSS, placed into 1.5 ml brown EP tubes, and immediately incubated with the probe at 28 °C in the dark for 30–60 min. The FerroOrange kit (F374, Ex: 561 nm, Em: 570–620 nm) was used for operations, and larvae were washed three times with 1 ml HBSS. Larvae were gently transferred to methylcellulose using a Pasteur pipette, and their position was fixed using an egg picker. Fluorescence microscopy parameters were fixed, and all embryos were imaged using identical imaging settings to detect fluorescence conditions and take photographs.

### 4.15 Transmission electron microscopy

Zebrafish larvae from different treatment groups were collected and fixed overnight at 96 hpf in 0.1 M PBS containing 2.5 % glutaraldehyde. PBS washes were performed three times for 5 min each. Samples were then fixed at room temperature in 0.1 M PBS solution containing 1 % osmium tetroxide for 2 h. Dehydration was performed using a series of ethanol gradients (25 %, 50 %, 75 %, 100 %). Samples were then incubated in acetone at room temperature for 20 min, followed by treatment with 50 %, 75 %, and 100 % epoxy resin for 1 h, 3 h, and overnight at 70 °C. Ultrathin sections were prepared using a Leica ultramicrotome (Germany) and stained with 3 % uranyl acetate and 3 % lead citrate. Observations were made using a transmission electron microscope operating at 120 KV.

### 4.16 Behavioral experiments

This experiment aimed to study changes in swimming behavior of zebrafish larvae from different groups. Materials used included a 12-well plate, temperature control equipment, lighting equipment, and the Viewpoint zebrafish tracking and activity recording system. First, different groups of zebrafish larvae were placed into 12-well plates, one per well, to ensure observation of individual behavioral changes across different groups. The experimental environment temperature was precisely set to 28 ± 0.5 °C to simulate their natural habitat and reduce stress responses. The light-dark alternating experimental design had a dark period of 90 min, following which light and dark alternated every 10 min. During this period, the Viewpoint zebrafish tracking system was used to monitor their movement trajectories in real time. The movement trajectory and position of the zebrafish were recorded every minute to obtain detailed data on their movement patterns.

### 4.17 Apoptosis detection

800 zebrafish larval samples were immediately placed into EP tubes containing cell separation fluid (mainly containing trypsin 0.2 %, papain 2 mg/mL, collagenase 0.3 mg/mL) for the preparation of cell suspensions. The obtained cell suspension was processed according to the instructions of the Annexin V-FITC/PI dual fluorescence apoptosis detection kit (Elabscience, China). Subsequent detection and analysis were performed using flow cytometry.

### 4.18 Prediction of transcription factor binding sites and dual-luciferase reporter gene detection

The DeepPBS online platform (https://deeppbs.usc.edu/) was used for the preliminary prediction of high-specificity DNA binding sequences of zebrafish *foxo1a* and human FOXO1.The upstream 2000 bp promoter sequence of the zebrafish *foxo1a* and human SLC7A11 were retrieved from NCBI and analyzed for the presence of *foxo1a* and FOXO1 high-specificity DNA binding sequences to mark predicted binding sites. Expression plasmids and reporter plasmids were constructed using pcDNA3.1 and pGL3-basic plasmids, respectively. pcDNA3.1 plasmids were linearized after double digestion, recovered, and purified. Truncated and full-length expression plasmids were constructed via homologous recombination using the ClonExpress® Ultra One Step Cloning Kit (Vazyme, China). Similarly, reporter plasmids (pGL ∼ slc7a11) were constructed by homologous recombination after circular pGL3-basic plasmid double digestion. The constructed plasmids were transformed into chemically competent Trans 1-T1 bacteriophage-resistant cells (TransGen, China). After monoclonal screening and bacterial culture, sequencing (Sangon, China) was performed. Bacterial strains with consistent amplification sequences were selected, and plasmids were extracted using an endotoxin-free plasmid extraction kit (TIANGEN, China). Human embryonic kidney 293T (HEK293T) cells frozen in liquid nitrogen were quickly revived in a 37 °C water bath and transferred to DMEM/high-glucose medium (Servicebio, China) containing 10 % fetal bovine serum (absin, China) for culture at 37 °C and 5 % CO_2_. Cells with confluency exceeding 95 % were passaged, and after normal stable growth for about 3–4 generations, cells were evenly distributed at a density of about 10^5^ cells/well in 24-well plates (CORNING, USA). When cell confluency reached 80 %, different truncated and full-length expression plasmids were transfected, and the aggregation of fluorescent droplets was observed to determine whether liquid-liquid phase separation occurred. When cell confluency reached 50 %∼70 %, the Xfect™ transfection kit (Takara, Japan) was used to transfect expression plasmids and reporter plasmids into the cells, while co-transfecting pRL-TK plasmids expressing Renilla luciferase (Promega, USA) to characterize transfection efficiency. A control group was set up in the experiment, with three technical replicates for each treatment. After 48 h of plasmid transfection, firefly and Renilla luciferase values were measured using a microplate reader (SYNERGY HTX Multi-Mode Reader, BioTek, USA) and dual-luciferase detection kit (Promega, USA).

### 4.19 Cloning of protein-coding sequences (CDS)

Using NCBI Primer BLAST, cDNA was used as a template to clone the CDS regions. The high-fidelity DNA polymerase reagent used was 2 × Phanta Max Master Mix (Dye Plus) (Vazyme, Nanjing), and the reaction system and program were set according to the product manual (for sequences with high GC content or special structures, the denaturation time in PCR cycles can be extended to 20–30 s). PCR products were subjected to 1 % agarose gel electrophoresis, and target DNA bands were excised and purified. The TOPO cloning method was used to ligate them into the pCE2 TA/Blunt Zero vector (Vazyme, Nanjing). The ligation product was transformed into competent Escherichia coli DH5α, and ampicillin-resistant plates were used to screen single colony clones for Sanger sequencing.

### 4.20 Total RNA extraction, reverse transcription, and RT-qPCR

Total RNA was extracted from different groups of zebrafish larvae using the TRIzol method. MonScriptTM RTIIII Super Mix with dsDNase (Two-Step) (Takara, Dalian, China) was used to remove gDNA and synthesize the first strand of cDNA, stored at −20 °C. qPCR primers were designed using NCBI Primer BLAST, with primer locations limited to the CDS region and qPCR product lengths limited to 80–200 bp. β-actin was used as an internal reference, and qPCR was used to detect the relative expression levels of related genes. The quantitative reagent used was MonAmp™ Fast SYBR® Green qPCR Mix (Takara, Dalian, China), and the reaction system and program were operated according to the manual. qPCR was performed on the ABI QuantStudio 3 system (Thermo Fisher Scientific, USA) to determine the cycle number required to reach the threshold (CT). Three technical replicates were set for each sample, and the 2^−ΔΔCT^ method was used to calculate relative expression levels.

### 4.21 Construction of overexpression vectors

Empty PCS2+ vectors were used to construct recombinant plasmids containing the CDS regions of *foxo1a* and *slc7a11* genes for microinjection into zebrafish embryos.

### 4.22 Statistical analysis

Each experiment was independently repeated at least six times. Data analysis was conducted using GraphPad Prism 10.1 software (GraphPad Software Inc., San Diego, CA, USA). Bar graph data are presented as mean ± standard deviation (mean ± SD). First, the normality assumption of the data was assessed using the Shapiro-Wilk test or the D'Agostino-Pearson omnibus test, and the homogeneity of variance assumption was evaluated using the Brown-Forsythe test. For comparisons between two groups: a Welch-corrected unpaired *t*-test was used. For comparisons among three groups: if the homogeneity of variance assumption was met, one-way analysis of variance (ANOVA) was applied, followed by Tukey's post hoc test. If the homogeneity of variance assumption was violated, Brown-Forsythe and Welch's ANOVA analyses were performed, with Dunnett's T3 test used for post hoc multiple comparisons. The asterisk notation is defined as follows: P > 0.05; ∗P < 0.01; ∗∗P < 0.01; ∗∗∗P < 0.001; ∗∗∗∗P < 0.001.

## CRediT authorship contribution statement

**Yinjie Zhao:** Data curation, Formal analysis, Writing – original draft, Writing – review & editing. **Zikang Li:** Data curation, Formal analysis, Writing – original draft, Writing – review & editing. **Weiqun Lu:** Funding acquisition, Methodology, Project administration, Supervision.

## Declaration of competing interest

The authors have declared that no conflict of interest exists.
