# Supplementary material for: Guardian of myelin and neural Integrity: foxo1a through slc7a11 mitigating oxidative damage in myelin
Source: Redox Biol. 2025 Jul 12;85:103763. doi: 10.1016/j.redox.2025.103763 (PMC12283898; doi:10.1016/j.redox.2025.103763)

Supplementary Information

Figure S1：

A: Analyze the fluorescence intensity of iron ions in the brains of wild-type and foxo1a-/- mutant zebrafish using ImageJ to determine their relative iron ion content. N=6, t-test, * $p < 0.05$, ** $p < 0.01$, *** $p < 0.001$, **** $p < 0.0001$

B: Analyze the fluorescence intensity of iron ions in the brains of zebrafish injected with empty vector mutants and those injected with foxo1a overexpression mutants using ImageJ to determine their relative iron ion content. MT represents foxo1a mutant zebrafish. N=6, t-test, * $p < 0.05$, ** $p < 0.01$, *** $p < 0.001$, **** $p < 0.0001$

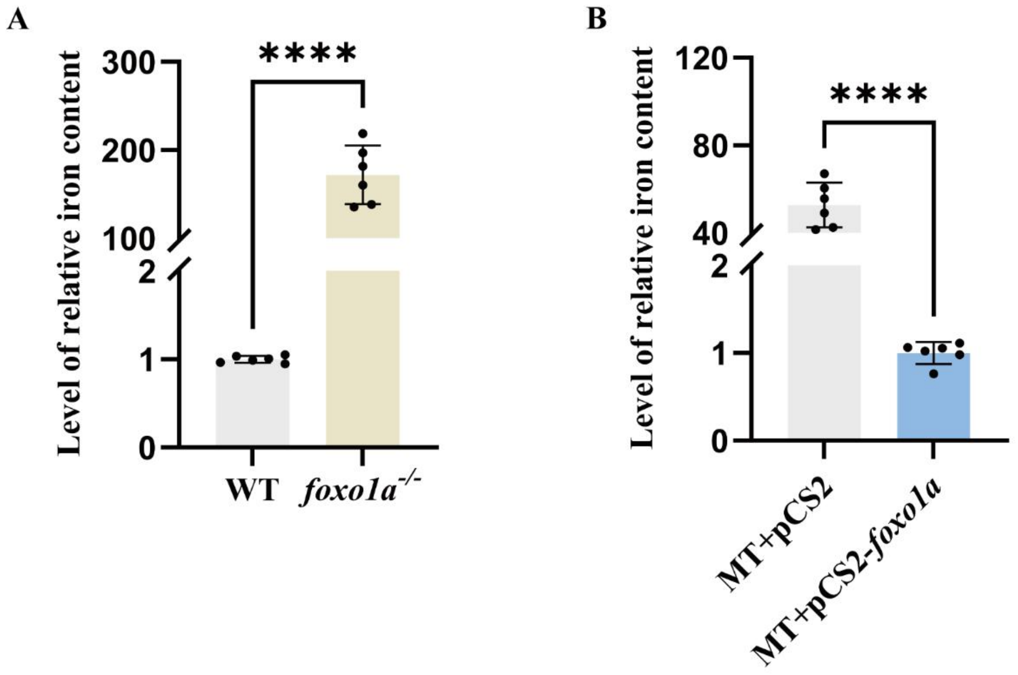

Figure S2：Expression of the *slc7a11* gene in embryos injected with negative control siRNA and *slc7a11*-siRNA four days after injection, as detected by qRT-PCR. N=6, t-test, * $p < 0.05$, ** $p < 0.01$, *** $p < 0.001$, **** $p < 0.0001$.

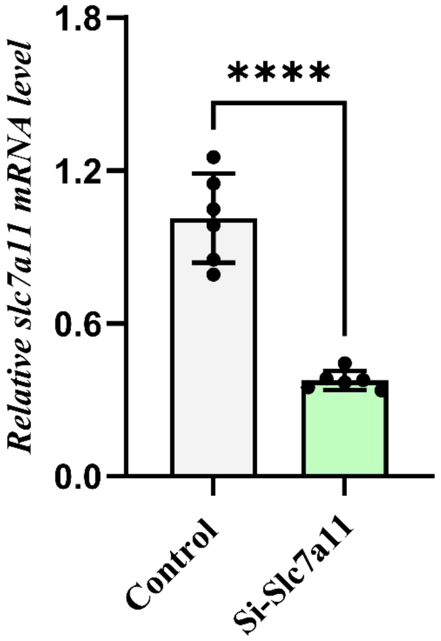

Supplement: Multimedia component 1 [file mmc1.pdf]
